# Antioxidant Activities of the Cell‐Free Supernatant of a Potential Probiotic *Cutibacterium acnes* Strain CCSM0331, Isolated From a Healthy Skin

**DOI:** 10.1111/jocd.70105

**Published:** 2025-03-12

**Authors:** Li Shao, Jieyan Huang, Yan Li, Laiji Ma, Yujie Niu, Wen Jiang, Chunying Yuan, Tianming Bai, Suzhen Yang

**Affiliations:** ^1^ School of Perfume and Aroma Technology Shanghai Institute of Technology Shanghai P. R. China; ^2^ R&D Innovation Center Shandong Freda Biotech Co., Ltd. Jinan Shandong P. R. China

**Keywords:** antioxidant, *Cutibacterium acnes*, human primary keratinocytes, Nrf‐2 signaling pathway, SCFAs, the reactive oxygen species (ROS)

## Abstract

**Objective:**

Oxidative stress activates the reactive oxygen species (ROS) and excessive ROS can damage skin cells, initiating oxidative stress responses that contribute to inflammation, aging, and other skin issues. As a resident skin bacterium, *Cutibacterium acnes* (*C. acnes*) plays an important role in maintaining skin homeostasis and provides antioxidant benefits. However, the metabolite components and mechanisms of *C. acnes* exerting antioxidant activity are not yet clear. This study aimed to analyze the potential antioxidant effects of *C. acnes* cell‐free supernatant and the mechanisms.

**Methods:**

The antioxidant effects were evaluated by measuring the scavenging activities of 2,2‐diphenyl‐1‐picrylhydrazyl (DPPH), 2,2′‐azinobis (3‐ethylbenzothiazoline‐6‐sulfonic acid ammonium salt) (ABTS) radicals, and hydroxyl radicals, as well as the effects on ROS levels in menadione‐induced primary human keratinocytes in vitro. Additionally, western blot analysis was performed to assess the antioxidant effects of the *C. acnes* CCSM0331 cell‐free supernatant (CFS).

**Results:**

*C. acnes* CCSM0331 was isolated from the facial skin of healthy individuals. This strain, classified as type II, is associated with healthy skin. The CFS of strain CCSM0331 contained various short‐chain fatty acids (SCFAs), glutathione peroxidase (GSH‐Px), and total superoxide dismutase(T‐SOD), exhibiting strong DPPH and ABTS radical scavenging capabilities, thus demonstrating substantial antioxidant activity. In a reactive oxygen species model induced by menadione in primary human keratinocytes, the addition of 5% of the fermentation supernatant from this strain significantly reduced ROS levels, indicating a notable ROS‐scavenging effect. Western blot analysis further confirmed that the CCSM0331 fermentation supernatant activated the expression of Nrf‐2 and HO‐1 proteins, thereby activating the Nrf‐2 oxidative stress pathway and exerting antioxidant effects.

**Conclusion:**

*C. acnes* CCSM0331 is a promising skin probiotic with notable antioxidant properties. The activity of this strain exhibited significant free radical scavenging activity, suggesting its potential application in the development of antiaging products. This study provides theoretical support for the screening of functional skin bacteria or skin probiotics.

## 1 Introduction

*Cutibacterium acnes* (*C. acnes*) is a commensal bacterium primarily in human hair follicles and sebaceous glands, as well as on the skin surface. *C. acnes* has gained notoriety due to its association with acne pathogenesis [1]. However, advances in genomics, proteomics, and metabolomics have significantly deepened our understanding of *C. acnes*. Recent studies have revealed that while the overall abundance of *C. acnes* does not differ significantly between healthy individuals and acne patients, there are notable differences in population structure at the strain level [2]. According to six housekeeping genes and two virulence genes, *C. acnes* was classified using a multilocus sequence typing (MLST) scheme into six phylogenetic types: IA1, IA2, IB, IC, II, and III. Notably, *C. acnes* isolated from healthy individuals and acne patients were distributed in distinct types, with IA1 being predominantly associated with acne and type II primarily isolated from healthy skin [3]. The discovery of these phylogenetic types has challenged the negative reputation of *C. acnes*, highlighting the critical role of the intricate balance between skin microbiome members and phylogenetic types of *C. acnes* in acne pathogenesis. Under normal physiological conditions, *C. acnes* functions as a commensal bacterium in humans. However, when the skin's physiological state changes or it relocates due to medical device usage, *C. acn*es may produce metabolites that trigger skin inflammation, exacerbate dermatological disorders, or contribute to systemic diseases [4, 5, 6].

Multiple studies have demonstrated a decline in the abundance of *C. acnes* with increasing age. The cheek skin of older individuals exhibited significantly lower levels of *C. acnes* compared to younger individuals [7, 8, 9], suggesting a potential association between *C. acnes* and a “youthful” skin phenotype. As we known, *C. acnes* plays a crucial role in maintaining skin health [10]. *C. acnes* hydrolyze sebum to produce free fatty acids, creating a mildly acidic environment that prevents the colonization of opportunistic pathogens [11]. The short‐chain fatty acids (SCFAs) produced by *C. acnes* not only inhibited the formation of *Staphylococcus epidermidis* biofilms [12] but also modulated keratinocyte lipid synthesis and altered the composition of epidermal lipids. These actions helped maintain the stability of the skin's commensal microbiome, thereby strengthening the skin's barrier function [13, 14]. Paetzold B et al. [15] isolated *C. acnes* from healthy individuals and developed a probiotic solution for topical application on healthy skin. This solution was shown to regulate human skin physiology while demonstrating safety. Increasing evidence supports the beneficial functions of *C. acnes*. Fournière et al. [16] proposed that *C. acnes* acted as a sentinel bacterium of the skin, playing a pivotal role in maintaining skin homeostasis. Screening beneficial *C. acnes* strains from healthy skin presents a promising opportunity for exploring skin probiotics.

Skin exposed to the external environment, particularly ultraviolet (UV) radiation, generates reactive oxygen species (ROS), disrupting the oxidative‐antioxidative balance within the skin. Excessive ROS triggers oxidative stress, leading to lipid, protein, and DNA damage, ultimately resulting in skin cell injury [17]. The skin microbiota, particularly the dominant species *C. acnes* and *S. epidermidis*, is continuously exposed to various environmental stresses, such as ROS generated by UV‐irradiated skin and toxic substances. These stresses impact their cellular metabolism and oxidative stress response systems [18, 19]. Studies have reported that *C. acnes* secreted a radical oxygenase (RoxP), which alleviated oxidative stress caused by ROS [18, 20]. *S. epidermidis* secreted butyric acid, an important SCFA, which reduced the expression of the inflammatory cytokine IL‐6 induced by ROS [21]. Skin microbiota and their metabolites play a vital role in maintaining oxidative stress homeostasis in the human body.

Increasing evidence suggests that *C. acnes* derived from different skin conditions exhibits variations in genotypes and virulence factors. Therefore, it is necessary to study *C. acnes* at the strain level. In our previous studies, many *C. acnes* strains were isolated from healthy skin, and DPPH radical scavenging activity was used as an indicator to screen the antioxidant level of the fermentation supernatants from different strains. Among these, *C. acnes* CCSM0331 exhibited excellent antioxidant properties [22]. This study aimed to investigate the fundamental biological characteristics, antioxidant effects, and antioxidant mechanisms of strain CCSM0331, providing a theoretical basis for the selection of functional skin bacteria or skin probiotics.

### 1.1 Bacterial strains, cell, culture, and media

*C. acnes* CCSM0331 was isolated from human facial skin, and *C. acnes* ATCC 6919 was purchased from GDMCC (Guangdong, China). Both bacterial strains were streaked on TSA (Hope Bio‐Technology Co. Ltd., Qingdao, China) plates for activation and incubated under anaerobic conditions at 37°C for 60–72 h, with activation performed twice. Single colonies from the second activation were selected and transferred into TSB (Hope Bio‐Technology Co. Ltd., Qingdao, China), followed by incubation under anaerobic conditions at 37°C for 72 h. The bacterial suspension was diluted with TSB to an optical density at 600 nm (OD_600_) of 0.3–0.4, measured using a Multiskan SkyHigh Microplate Spectrophotometer (Thermo Fisher Scientific, MA, USA). Human primary skin keratinocytes were purchased from Lifeline Cell Technology (California, USA) and cultured in a complete culture medium (Promocell, Germany). All cells were cultured in a humidified atmosphere of 5% CO_2_ at 37°C.

### 1.2 Chemicals and materials

Tween‐20 was purchased from Shanghai Titan Technology Co. Ltd. (Shanghai, China). DPPH (2,2‐diphenyl‐1‐picryl‐hydrazyl) was purchased from Yuanye Biotechnology Co. Ltd. (Shanghai, China). ABTS (2,2′‐Azinobis (3‐ethylbenzothiazoline‐6‐sulfonic Acid Ammonium Salt)) was purchased from Shanghai Aladdin Biochemical Technology Co. Ltd. (Shanghai, China). An SCFA‐mixed standard solution, comprising acetic acid, propionic acid, butyric acid, isobutyric acid, valeric acid, and isovaleric acid, was purchased from Northern Weiye Metrology Research Institute (Beijing, China).

## 2 Experimental Methods

### 2.1 Strain Screening

Healthy skin volunteers were recruited, and sterile cotton swabs moistened in a wetting solution (0.9% sodium chloride and 0.1% Tween‐20) were used to swab selected facial areas. The swabs were placed in 5 mL of sterile water in centrifuge tubes, mixed thoroughly, and serially diluted. 0.1 mL of the dilution was plated onto TSA blood agar plates and incubated anaerobically at 37°C for 72 h. Single colonies were selected for multiple streaks to obtain pure cultures, and the colony morphology on the plates was observed.

### 2.2 Strain Identification

A single purified colony of CCSM0331 was inoculated into 10 mL of TSB medium and incubated anaerobically at 37°C for 60–72 h. The bacterial culture was centrifuged at 8000 r/min for 15 min to collect the cells. DNA was extracted using a DNA extraction kit (Sangon Biotech, Shanghai, China). PCR amplification was performed using two synthesized universal primers (16S 27F: GAGAGTTTGATCCTGGCTCAG; 16S 1473R: CGGCTACCTTGTTACGACTT). PCR products were recovered using a gel extraction kit (Bio Flux), purified, and sent to Majorbio (China) for sequencing. Sequencing results were aligned using BLAST in the NCBI database to identify the species of the strain.

### 2.3 Observation of Bacterial Morphology

The bacterial morphology of *C. acnes* CCSM0331 was observed by scanning electron microscopy (SEM) according to the method of Wang et al. [23], with slight modifications. The bacterial suspensions of *C. acnes* CCSM0331 were diluted to 1 × 10^8^ CFU/mL in a fresh TSB medium. Bacterial suspensions were inoculated into a TSB medium at 1% (v/v) and incubated anaerobically at 37°C until mid‐logarithmic growth. The bacteria were washed with PBS and then centrifuged at 1000 × g for 10 min at 4°C to collect the bacterial cells. The cells were fixed overnight in 2.5% glutaraldehyde, then washed with PBS, dehydrated using a series of ethanol gradients (50%, 70%, 80%, 95%, 100%), and replaced with isoamyl acetate. The samples were freeze‐dried, gold‐coated, and observed and photographed using a Gemini 300 scanning electron microscope (ZEISS, Germany).

### 2.4 Determination of Growth Curve

The activated *C. acnes* CCSM0331 was inoculated into TSB medium at a 2% (v/v) inoculum ratio and incubated anaerobically at 37°C without shaking for 96 h. The optical density (OD) at 600 nm was measured every 6 h to construct a growth curve of CCSM0331 in a TSB medium. Additionally, the pH of the culture medium was measured at each growth curve time point.

### 2.5 Determination of adhesion curve

The bacterial suspension was prepared to a concentration of 1 × 10^7^ CFU/mL in TSB medium. A total of 150 μL of TSB medium and 50 μL of bacterial suspension were added to each well of a 96‐well plate. The plates were incubated anaerobically at 37°C for 144 h. Every 24 h, one plate was removed, the liquid discarded, and the wells washed three times with 0.9% saline. A 0.01% crystal violet solution was added to the dried wells for 15 min of staining, followed by two washes with sterile deionized water. The plates were dried, and the fixed crystal violet was released using 95% ethanol. Absorbance was measured at 595 nm. OD_595_ values were plotted over time, with *C. acnes* ATCC6919 (nonbiofilm forming) used as a control to construct the adhesion curve of *C. acnes* CCSM0331.

### 2.6 Preparation of Fermentation Supernatant

The seed culture was inoculated into 100 mL triangular flasks at an inoculum ratio of 1%–3% (v/v) and incubated anaerobically at 37°C without shaking for 72 h to obtain the fermentation broth of *C. acnes* CCSM0331. The fermentation broth was centrifuged at 9,000 r/min for 10 min, and the supernatant was filtered through a 0.22 μm membrane to obtain the fermentation supernatant of *C. acnes* CCSM0331.

### 2.7 DPPH Radical Scavenging Assay

Free radical scavenging activity of methanol extract was determined using the 1,1‐diphenyl‐2‐picrylhydrazyl (DPPH) method [24]. One milliliter of DPPH working solution was mixed with 1 mL of CCSM0331 fermentation supernatant at different concentrations, and water was added to adjust the final volume to 4 mL. The mixtures were incubated in the dark at room temperature for 30 min, and the absorbance was measured at 517 nm. Each sample was tested in triplicate, and water was chosen to a final volume of 4 mL and used as a control. The activity of scavenging (%) was calculated using the following formula: DPPH radical scavenging % = [(OD control−OD sample)/OD control] × 100.

### 2.8 ABTS Radical Scavenging Assay

The ABTS assay was conducted as described in Rumpf et al. [25]. The diluted fermentation supernatant of *C. acnes* CCSM0331 was mixed with ABTS^+^ working solution to a final volume of 4.0 mL and incubated in the dark for 5 min. Absorbance was measured at 734 nm. A mixture of 1.0 mL ABTS^+^ working solution and 3.0 mL anhydrous ethanol was prepared to a final volume of 4.0 mL and used as the blank control. Vitamin C (V_C_) at the same mass concentration was used as a positive control. Each sample was tested in triplicate. ABTS scavenging (%) = [(OD control—OD sample) / OD control] × 100.

### 2.9 Hydroxyl radical scavenging assay

A total of 200 μL of the fermentation supernatant of *C. acnes* CCSM0331 was used to measure hydroxyl radical (OH·.^−^) scavenging activity according to the instructions of the hydroxyl radical assay kit (Nanjing Jiancheng Bioengineering Institute).

### 2.10 Determination of antioxidant enzyme activity

A total of 100 μL of the fermentation supernatant of *C. acnes* CCSM0331 was used to measure the activity levels of glutathione peroxidase (GSH‐Px), total superoxide dismutase (T‐SOD), and catalase (CAT) according to the instructions of the corresponding assay kits (Nanjing Jiancheng Bioengineering Institute).

### 2.11 Determination of the SCFAs content

The SCFAs content was determined using gas chromatography (GC) according to the method described by Shao et al. [26]. 2 mL of fermentation supernatant was filtered through a 0.22 μm filter, mixed with 0.4 mL of 50% H_2_SO_4_ and 2 mL of ether, and shaken at 250 r/min for 45 min. The mixture was then centrifuged at 4°C and 10,000 × g for 5 min, and the upper organic phase was collected. A stock solution of 1000 μg/mL was prepared by diluting the mixed standard solution with ultrapure water, and gradient samples (5, 10, 20, 50, 100, 200, 500, 800, and 1000 μg/mL) were prepared. The GC conditions were as follows: an injection volume of 1 μL, starting from 75°C and ramping to 180°C at 10°C/min, holding for 1 min, then ramping to 220°C at 10°C/min and holding for 1 min. The analysis was repeated three times, and the average value was obtained. Acetic acid, isovaleric acid, propionic acid, butyric acid, and pentanoic acid were used as standards.

### 2.12 Determination of the viability of human primary keratinocytes

Human primary skin keratinocytes were cultured in DMEM high‐glucose complete medium (Lifeline Cell Technology) under conditions of 5% CO_2_ and 37°C. When the cells reached 80%–90% confluence, they were digested with trypsin and seeded into 96‐well plates at a density of 1 × 10^5^ cells/mL. After 48 h of adhesion, the cells were treated with various concentrations of 4× concentrated fermentation supernatant of *C. acnes* CCSM0331 for 24 h. Each experimental group included three parallel wells. The cells were washed once with DPBS, and cell viability was measured using a CCK‐8 assay kit (Dojindo Laboratories, Japan) at 450 nm. Cell viability (%) = (OD of the sample group/OD of the control group) × 100%.

### 2.13 ROS induction assay in human primary skin keratinocytes

Human primary skin keratinocytes were cultured as described above. Upon reaching 80%–90% confluence, they were seeded into black‐walled 96‐well plates at 1 × 10^5^ cells/mL. After 48 h of adhesion, cells were treated with different concentrations of samples containing 10 μM menadione (Sigma‐Aldrich, USA) for 24 h. Vitamin E (Solarbio, Beijing, China) was used as a positive control. After discarding the supernatant, the cells were incubated with ROS fluorescent dye for 1 h. Fluorescence intensity was measured using a microplate reader (excitation 485 nm, emission 520 nm).

### 2.14 Nrf2 protein pathway assay

Human primary skin keratinocytes were cultured as described above and seeded into 6‐well plates at 5 × 10^6^ cells/mL. After 48 h, cells were treated with 5% fermentation supernatant for 24 h. Cytoplasmic and nuclear proteins were extracted, separated by 10% SDS‐PAGE, and transferred to a PVDF membrane. The membrane was blocked with 5% skimmed milk for 2 h, then incubated with primary antibodies overnight at 4°C, followed by secondary antibodies for 1 h at room temperature. Protein expression levels of Nrf2 and HO‐1 were analyzed using ImageJ software, with Lamin B as an internal reference.

### 2.15 Statistical data analysis

Statistical significance was assessed using one‐way analysis of variance (ANOVA) followed by Tukey's multiple comparisons test, conducted using SPSS version 26.0. *p < *0.05*, **p < 0.01, and ***p < 0.0001 indicate statistically significant differences.

## 3 Results and Analysis

### 3.1 Morphological and biological characteristics of strain CCSM0331

**FIGURE 1:**
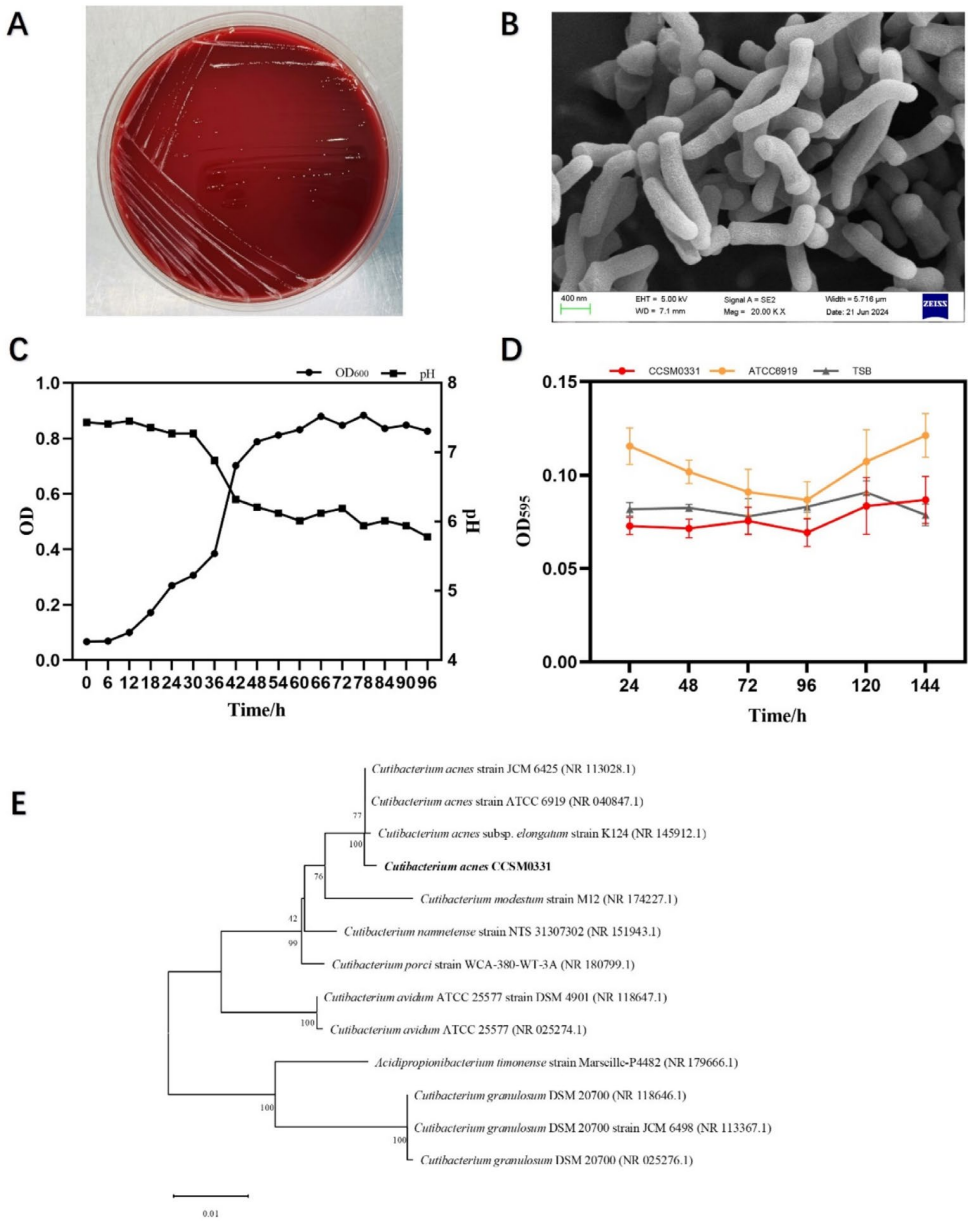
Basic biological characteristics of *C. acnes* CCSM0331 (A) TSA blood agar colony morphology (B) Scanning electron microscopy (SEM) (C) Growth curve (D) Adhesion curve (E) Phylogenetic tree.

Strain CCSM0331 was isolated from the facial skin of a healthy individual. When cultured anaerobically on TSA blood agar plates for 72 h, the colonies of strain CCSM0331 appeared as slender rods, approximately 0.5 mm in size, with a shiny surface and no hemolytic zone (Figure 1A). Scanning electron microscopy revealed rod‐shaped cells arranged in X, Y, or V configurations (Figure 1B). When cultured in a TSB medium, the growth curve and pH curve (Figure 1C) indicated that strain CCSM0331 entered the early exponential phase at 18 h. Between 18 and 66 h, the growth rate significantly accelerated, entering the logarithmic phase, and transitioned to the stationary phase after 78 h. Semiquantitative crystal violet staining was used to assess biofilm formation, with *C. acnes* ATCC6919 as a control strain. The adhesion curve (Figure 1D) indicated that strain CCSM0331 did not form biofilms. A phylogenetic tree based on the 16S rRNA gene sequence (Figure 1E) showed that strain CCSM0331 shares the highest homology (99.19%) with strain 145912.1. Whole‐genome sequencing revealed that the genome of this strain is 2,496,923 bp in length, with an average GC content of 60.02%. The strain lacked plasmids and was classified as Type II (NCBI accession number: CP114143) [22]. This strain has been deposited in a culture collection center with the accession number CCTCC No. M2022781.

### 3.2 In vitro antioxidant activity of fermentation supernatant

**FIGURE 2:**
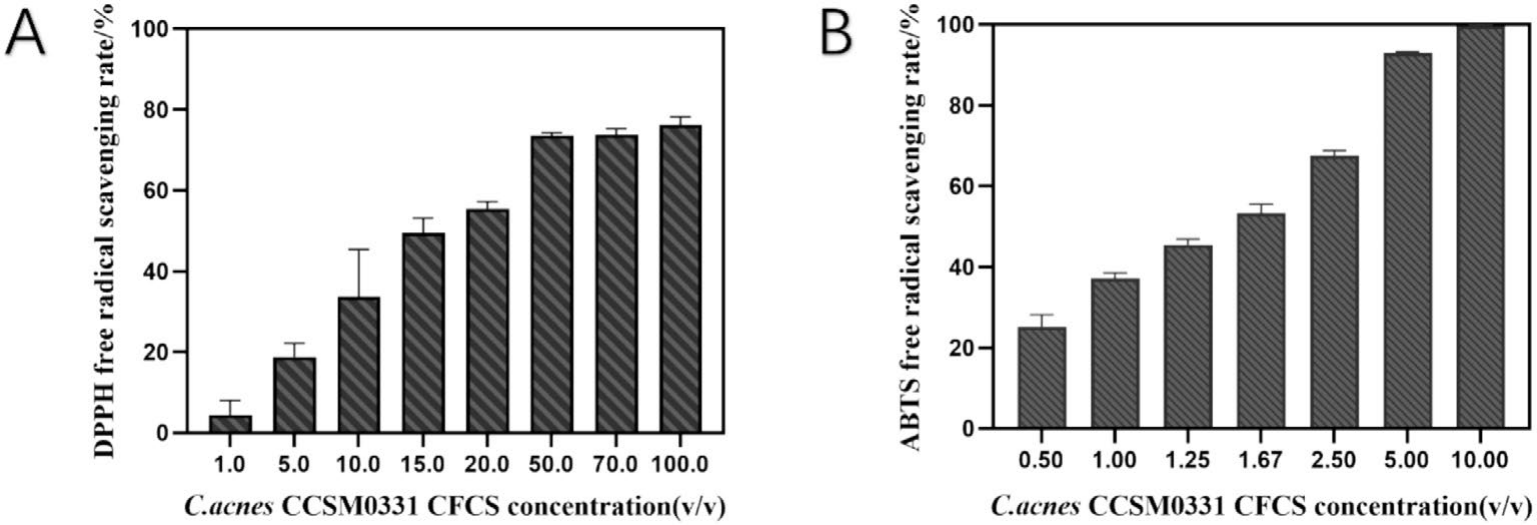
The scavenging effects of *C. acnes* CCSM0331 fermentation supernatant on DPPH radicals and ABTS^+^ radicals.

The fermentation supernatant of CCSM0331 was evaluated for its scavenging activity against DPPH radicals, ABTS^+^ radicals, and hydroxyl radicals. As shown in Figure 2, the scavenging effect on DPPH and ABTS^+^ radicals increased with the concentration of the fermentation supernatant. The undiluted supernatant exhibited scavenging rates of 76.20% and 99.56% for DPPH and ABTS^+^ radicals, respectively, and 5.34 ± 0.01 U/mL for hydroxyl radicals. The IC_50_ values for scavenging DPPH and ABTS^+^ radicals were calculated to be 18.58% and 1.38%, respectively, indicating that the fermentation supernatant of *C. acnes* CCSM0331 possesses strong antioxidant activity.

### 3.3 Antioxidant Enzyme Activity and Short‐Chain Fatty Acid Content

Antioxidant enzymes play a crucial role in protecting the body from oxidative damage induced by ROS. The fermentation supernatant of CCSM0331 contained GSH‐Px and T‐SOD enzyme activities of 14.38 ± 4.35 U/mL and 8.08 ± 0.88 U/mL, respectively, whereas CAT activity was undetectable. T‐SOD and GSH‐Px were primarily present in the cell‐free supernatant, whereas CAT activity, predominantly associated with cell surface components, was low. GC analysis determined the types and concentrations of SCFAs in the CCSM0331 fermentation supernatant, as shown in Table 1. The primary SCFAs were propionic acid, acetic acid, and isovaleric acid, which accounted for 97% of the total SCFAs. Minor amounts of isobutyric acid, butyric acid, and valeric acid were also detected. The total concentration of SCFAs was 2583.27 μg/mL.

**TABLE 1:** The concentration and proportion of short‐chain fatty acids in the fermentation supernatant of strain CCSM0331.

CCSM0331 supernatant	Concentration (μg/mL)	Proportion (%)
Acetic acid	379.74	14.70
Propionic acid	1991.70	77.10
Isobutyric acid	10.07	0.39
Butyric acid	13.69	0.53
Isovaleric acid	172.05	6.66
Valeric acid	16.02	0.62
Total short‐chain fatty acids	2583.27	100

### 3.4 Cytotoxicity to skin keratinocytes

**FIGURE 3:**
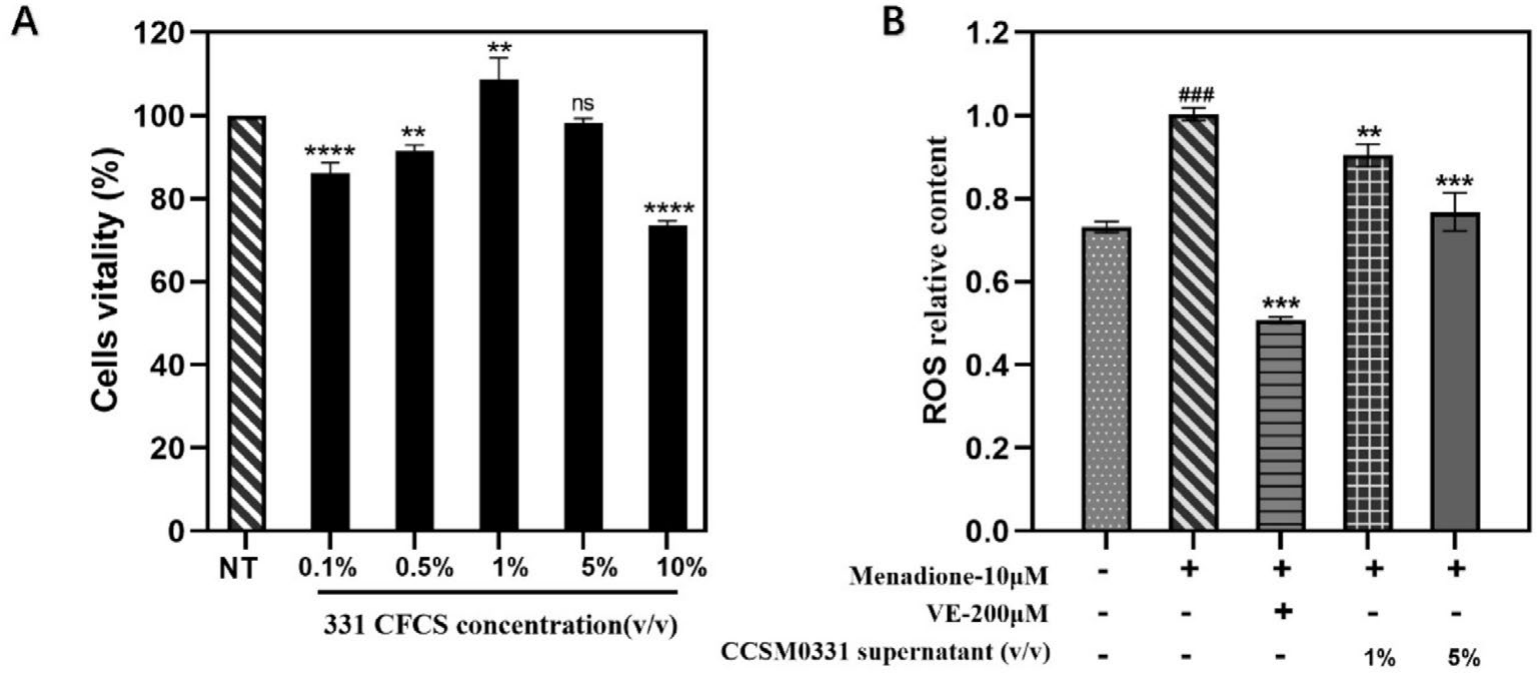
Effects of CCSM0331 fermentation supernatant on cell viability and ROS levels in human primary keratinocytes. (A) Cell viability of human primary keratinocytes treated with different concentrations of CCSM0331 fermentation supernatant was evaluated. The results showed that the 1% and 5% concentrations did not exhibit cytotoxic effects, while the 10% concentration significantly reduced cell viability. (B) ROS levels in human primary keratinocytes after menadione‐induced oxidative stress were measured. Treatment with 1% and 5% CCSM0331 fermentation supernatant reduced ROS levels to 90.38% (**p < 0.01) and 78.61% (***p < 0.0001), respectively, compared to the menadione group. Vitamin E (VE, 200 μM) was used as a positive control, reducing ROS levels to 50.73% (***p < 0.0001).

The fermentation supernatant of CCSM0331 was collected and co‐cultured with human primary keratinocytes at various concentrations to assess cell viability. As shown in Figure 3A, the fermentation supernatant of CCSM0331 at a 10% concentration exhibited cytotoxic effects, whereas 1% and 5% concentrations showed no cytotoxicity. Additionally, the 10% TSB solution was nontoxic. According to the experimental results, 1% and 5% concentrations of CCSM0331 fermentation supernatant will be used in subsequent ROS inhibition assays to evaluate antioxidant efficacy.

### 3.5 Scavenging ROS‐free radicals

Based on the cell viability results, 1% and 5% concentrations of CCSM0331 fermentation supernatant were selected for ROS induction assays, with vitamin E (V_E_, 200 μM) serving as the positive control group. The ROS scavenging results are shown in Figure 3B. ROS levels in human primary keratinocytes significantly increased after induction with menadione, while treatment with vitamin E (V_E_, 200 μM) suppressed ROS induction, reducing the relative ROS content to 50.73% (*p* < 0.001). These results confirmed the reliability of the negative and positive controls, validating the experimental system. Treatment with 1% and 5% concentrations of CCSM0331 fermentation supernatant for 24 h showed inhibitory effects, with relative ROS contents of 90.38% (*p* < 0.01) and 78.61% (*p* < 0.001), respectively.

### 3.6 Nrf2 protein pathway

**FIGURE 4:**
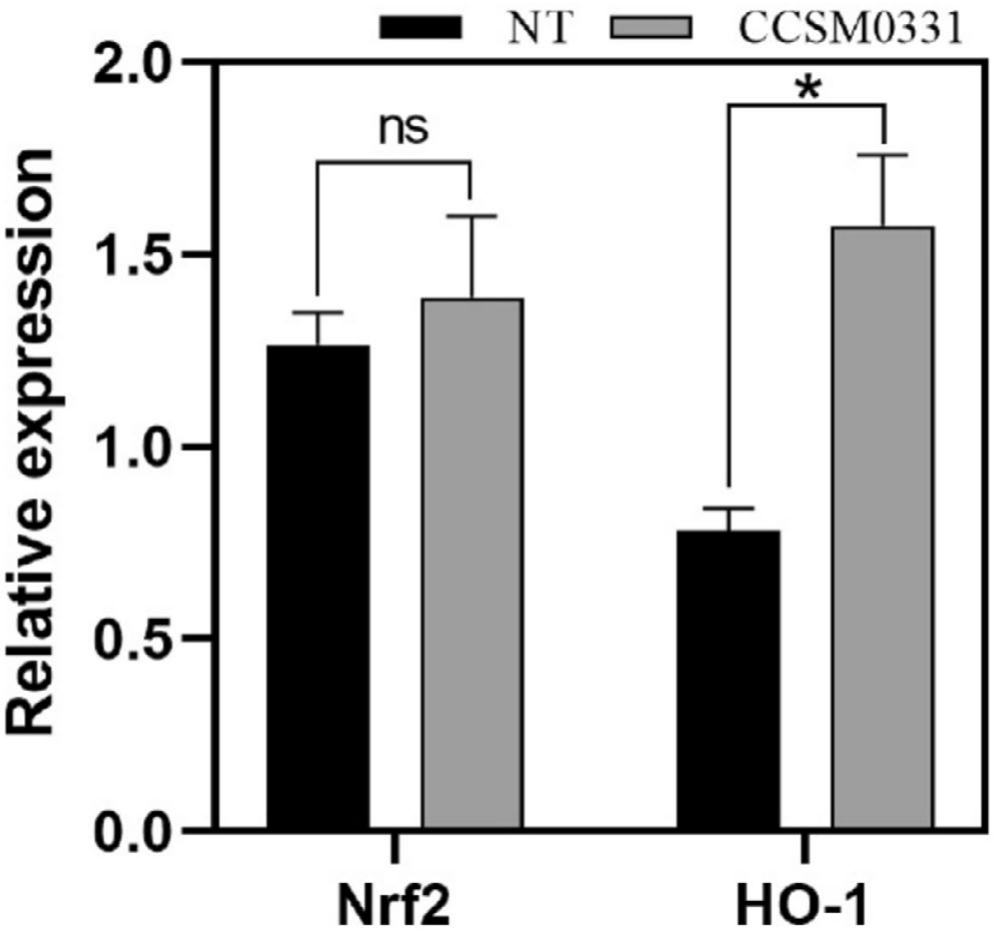
Effects of CCSM0331 fermentation supernatant on Nrf2 and HO‐1 protein expression.

Nuclear factor erythroid 2‐related factor 2 (Nrf2) is an essential transcription factor involved in cellular antioxidant responses and is considered a central component of the antioxidant signaling pathway. Under oxidative stress, excessive ROS triggers the dissociation of Nrf2 from Keap1, allowing Nrf2 to translocate into the nucleus. This increases Nrf2 protein expression and activates the oxidative stress pathway, upregulating the downstream target protein HO‐1. Using Lamin B as the internal reference, the addition of 5% fermentation supernatant of strain CCSM0331increased Nrf2 protein expression (*p* > 0.05) and significantly enhanced HO‐1 protein expression (*p* < 0.05) in Figure 4, thereby eliminating excessive ROS. The CCSM0331 fermentation supernatant activated the Nrf2 signaling pathway, significantly promoting HO‐1 expression and enhancing the cell's ability to mitigate excessive ROS.

The 5% CCSM0331 fermentation supernatant increased Nrf2 expression (*p >* 0.05) and significantly enhanced HO‐1 expression (*p < 0.05) in human primary keratinocytes. Lamin B was used as the internal reference. Error bars represent the standard deviation (SD).

## 4 Discussion

In recent years, an increasing number of studies have demonstrated that the skin microbiota plays a crucial role in maintaining skin homeostasis [27, 28]. The beneficial metabolites of skin commensal microbiota, isolated from healthy skin, can be used as cosmetic raw materials. For example, one Korean study collected *Lactobacillus plantarum* from 20s women and isolated its extracellular vesicles to apply to the face of the elderly [29]. *Staphylococcus hominis* , isolated from healthy individuals, has been shown to alleviate symptoms of atopic dermatitis (AD) [30]. *Streptococcus*, isolated from youthful skin, secreted spermidine, which helped to delay skin aging [31]. As for *C. acnes*, many studies have confirmed it could be a potential probiotic. *C. acnes* produced an antibiotic against *Staphylococcus aureus* , and its fermentation could inhibit methicillin‐resistant *S. aureus* , which is a pathogen commonly found in patients with AD [30, 32, 33]. *Staphylococcus* species are known to cause medical device‐associated infections, and biofilm production is one of their main virulence factors. The cell‐free conditioned media (CFCM) of *C. acnes* reduced biofilm formation without impacting their planktonic growth [12, 34]. These findings suggested that *C. acnes* can inhibit or reduce the virulence of skin‐pathogenic bacteria. Additionally, propionic acid produced during the fermentation of *C. acnes* reduced UV‐B‐induced melanin synthesis; it may be a natural acidic formulation to reduce skin pigmentation. What is most important is that the fermentation did not alter melanocyte proliferation and caused no cellular damage [35]. Recent studies have shown that *C. acnes* supports skin health through various mechanisms. It produces SCFAs that strengthen the skin barrier, reduce inflammation, and enhance essential lipid levels. These effects contribute to skin homeostasis by providing antioxidant activity [13, 36]. Based on the association between skin aging and the decreased abundance of *C. acnes*, we hypothesize that *C. acnes* could serve as a potential antiaging ingredient for cosmetic development.

The skin is constantly exposed to environmental factors, such as UV radiation and pollution, which generate free radicals. Free radicals attack skin cells, leading to cellular damage and aging. Free radical‐induced damage manifests as visible signs of skin aging, such as wrinkles, sagging, and hyperpigmentation. Free radicals can trigger inflammatory responses, resulting in skin redness, swelling, and itching [37]. The skin microbiota, as a biological barrier, plays a protective role against oxidative damage, particularly *C. acnes*, which resides on the skin. Multiple *C. acnes* strains were isolated from healthy skin, with DPPH radical scavenging activity used as an indicator; the strain CCSM0331 with the strongest antioxidant effect was identified. Its antioxidant properties and mechanisms were further analyzed. Our study provided a theoretical foundation for the development of probiotics or microbiota‐derived ingredients for skincare products.

Based on housekeeping gene typing analysis, the *C. acnes* strain CCSM0331 belongs to type II, which is associated with healthy skin [22], differing from acne‐prone types [3]. The adhesion curve of strain CCSM0331 indicated that this strain produced low levels of biofilm. CCSM0331, isolated from healthy skin, demonstrated safety advantages and showed potential as a beneficial skin bacterium.

SCFAs play a crucial role in lowering skin pH, reducing inflammation, inhibiting harmful bacterial colonization, and maintaining skin health [38, 39, 40]. *C. acnes* CCSM0331 produced various SCFAs, including propionic acid, acetic acid, isovaleric acid, and butyric acid. Propionic acid, accounting for 70% of the total, plays a key role in maintaining the skin's acidic pH, forming an acidified sebum film, and reducing pathogenic bacterial colonization. Shu et al. [33] found that *C. acnes* fermented glycerol to produce SCFAs, primarily propionic acid, isobutyric acid, and isovaleric acid, which helped establish ecological niche dominance. These SCFAs lowered intracellular pH levels in the skin, inhibited the growth of acid‐intolerant bacteria in the pilosebaceous unit, and reduced epidermal damage and inflammation caused by *S. aureus* . Additionally, some metabolites exhibited direct antibacterial effects. Propionic acid, a metabolite of *C. acnes*, is a well‐known antimicrobial SCFA [41, 42]. These SCFAs could also reduce the antibiotic resistance of *S. epidermidis* by inhibiting its biofilm formation [12]. The balance of the skin microbiota is crucial for maintaining the skin's antioxidant capacity. When this balance is disrupted, the overgrowth of harmful bacteria may lead to excessive ROS production, exacerbating oxidative stress in the skin [43]. *C. acnes* CCSM0331 produced various SCFAs that inhibit the colonization of harmful bacteria and the excessive production of ROS, helping to maintain the balance of the skin microbiota and support its antioxidant capacity.

Microorganisms produce antioxidant enzymes, such as T‐SOD, GSH‐PX, and CAT, which can eliminate ROS and mitigate oxidative stress‐induced damage to the body [44, 45]. Different *Lactobacillus* strains produce various antioxidant enzymes through metabolism, with numerous reports highlighting their antioxidant effects [46]. However, research on the secretion of oxidases by skin microbiota remains limited. Our results indicated that CCSM0331 metabolically produced antioxidant enzymes such as SOD and GSH‐Px, which could effectively scavenge free radicals and alleviate oxidative stress‐induced skin damage. Comparative genomic analysis revealed the presence of the Roxp enzyme gene in CCSM0331, which may partly explain its strong antioxidant properties, although its expression level was not quantified in this study.

Nrf2 serves as a central regulator of the body's antioxidant defense system, playing a crucial role in cellular responses to oxidative stress, and is widely acknowledged as a primary antioxidant signaling pathway [47, 48]. The fermentation supernatant of *C. acnes* CCSM0331 activated the Nrf2 pathway, leading to increased expression of its downstream target protein HO‐1, which is a key mechanism for its antioxidant effects.

Our results demonstrated that *C. acnes* CCSM0331 exerted its antioxidant effects through multiple mechanisms, including the metabolic production of antioxidant enzymes, short‐chain fatty acids, and activation of ROS antioxidant pathways. *C. acnes* CCSM0331 not only helped to maintain skin pH stability but also ensured skin microbiota and skin homeostasis, protecting against oxidative damage caused by environmental stressors and contributing to skin health maintenance.

Skin microbiota plays a crucial role in maintaining the balance of the skin microecosystem and overall skin homeostasis [49, 50]. For the diagnosis and treatment of skin diseases such as AD, skin microbiota has been incorporated into treatment guidelines [51]. Experts have also proposed microbiome‐derived solutions in skin health and care [52]. As a dominant skin bacterium and sentinel species, *C. acnes*, isolated from healthy skin, has the potential to serve as a beneficial skin bacterium for improving skin health in the near future. Currently, live bacteria cannot yet be used in skincare product development. However, CCSM0331, isolated from healthy skin, demonstrates strong antioxidant effects through its fermentation supernatant and can be applied as a postbiotic in skincare formulations.

The application of postbiotics of potential beneficial skin bacteria in functional cosmetics has become a possibility. This not only introduces a novel skincare concept and product development approach but also establishes a new field for studying skin microbiota and their biological functions in promoting human health. The development and utilization of *C. acnes* derived from healthy skin can make up the current lack of functional skin bacterial resources. It holds significant research value in exploring the probiotic potential and functional mechanisms of commensal *C. acnes*.

## 5 CONCLUSIONS

*C. acnes* CCSM0331, isolated from the facial skin of healthy individuals, is classified as type II, which is associated with healthy skin. The fermentation supernatant of strain CCSM0331 contained various SCFAs and antioxidant enzymes such as GSH‐Px and T‐SOD. It demonstrated excellent scavenging effects on DPPH, ABTS, and hydroxyl radicals, significantly reducing ROS levels in primary human keratinocytes. The strain exerts its antioxidant effects through multiple mechanisms, including the metabolic production of antioxidant enzymes, SCFAs, and activation of ROS antioxidant pathways. *C. acnes* CCSM0331 contributed to maintaining skin pH stability, ensuring skin and skin microbiota homeostasis, and protecting against oxidative damage caused by environmental stressors, thereby promoting skin health. *C. acnes* CCSM0331 is a potential probiotic strain with strong antioxidant effects, playing a vital role in oxidative stress regulation and maintaining skin health. The fermentation supernatant of *C. acnes* can be utilized as a postbiotic in the development of antiaging products. Our study provided a theoretical foundation for the screening of functional skin microbiota or probiotics.

## Author Contributions

Li Shao, Jieyan Huang, and Yujie Niu: Data curation and writing – original draft; Yan Li and Laiji Ma: Supervision; Wen Jiang, Chunying Yuan, Tianming Bai, and Suzhen Yang: Writing – review and editing.

## Conflicts of Interest

The authors declare no conflicts of interest.

## Data Availability

The data that support the findings of this study are available from the corresponding author upon reasonable request.
